# Influence of Companion Planting on Microbial Compositions and Their Symbiotic Network in Pepper Continuous Cropping Soil

**DOI:** 10.4014/jmb.2211.11032

**Published:** 2023-03-31

**Authors:** Jingxia Gao, Fengbao Zhang

**Affiliations:** Institute of Soil and Water Conservation, Northwest A&F University, Yangling 712100, P.R. China

**Keywords:** Continuous cropping, companion planting, soil enzyme, microbial diversity, symbiotic network

## Abstract

Continuous cropping obstacles have become a serious factor restricting sustainable development in modern agriculture, while companion planting is one of the most common and effective methods for solving this problem. Here, we monitored the effects of companion planting on soil fertility and the microbial community distribution pattern in pepper monoculture and companion plantings. Soil microbial communities were analyzed using high-throughput sequencing technology. Companion plants included garlic (T1), oat (T2), cabbage (T3), celery (T4), and white clover (T5). The results showed that compared with the monoculture system, companion planting significantly increased the activities of soil urease (except for T5) and sucrase, but decreased catalase activity. In addition, T2 significantly improved microbial diversity (Shannon index) while T1 resulted in a decrease of bacterial OTUs and an increase of fungal OTUs. Companion planting also significantly changed soil microbial community structures and compositions. Correlation analysis showed that soil enzyme activities were closely correlated with bacterial and fungal community structures. Moreover, the companion system weakened the complexity of microbial networks. These findings indicated that companion plants can provide nutrition to microbes and weaken the competition among them, which offers a theoretical basis and data for further research into methods for reducing continuous cropping obstacles in agriculture.

## Introduction

Pepper, a plant in the genus *Capsicum* of the Solanaceae family, is a widely cultivated vegetable in Ningxia, China. At present, improving the yield and quality of pepper has become a topic of interest as it is rich in capsaicin, ascorbic acid, vitamin C, and other nutrients, and exerts antibacterial and antioxidant functions [1]. In recent years, with the large-scale and programmed development of agricultural facilities, pepper planting bases have become more concentrated, and continuous cropping phenomenon is increasingly common, which resulted in imbalances in soil nutrients and microbial community structure, as well as a series of other problems, such as secondary salinization, crop root exudate and salt accumulation, aggravation of soilborne diseases, and deterioration of the ecological environment [2, 3]. These adverse conditions represent serious obstacles to continuous cropping and have led to reduced crop quality and yield, increased soilborne diseases, and restriction on the sustainable development of the pepper industry.

As an effective way to solve continuous cropping obstacles, companion planting is not only one of the most common planting methods in modern agriculture, but also an important biological control measure [4]. Companion planting involves planting different types of plants in close proximity at the same time, which not only contributes to pest control and pollination, but also optimizes nutrient supply and maximizes space utilization [4]. Moreover, companion planting plays an important role in improving soil available nutrients, enhancing soil enzyme activities, enriching the diversity of microbial species, and reducing the occurrence of soilborne diseases [5-7]. When wheat is accompanied with white lupin, citric acid secreted by white lupin roots can activate soil phosphorus, further improving the phosphorus absorption of wheat [8]. Similarly, the combination of green onion and cucumber can improve the potassium absorption of cucumber plants [9].

A large number of studies have shown that using the principle of allelopathy between plants and reasonably arranging companion plants or intercropping not only improves the yield and quality of vegetables, but also effectively reduces diseases and pests and improves the soil ecological environment to reduce the occurrence of continuous cropping obstacles [10, 11]. Cowpea/sorghum intercropping and peanut/corn intercropping activated soil phosphorus and increased soil phosphatase activity [12, 13]. Studies have found that the root exudates of companion plants regulate the soil microecological environment by inhibiting the growth of pathogenic microorganisms and further promoting seedling and plant growth [14]. Additionally, companion planting promotes advantageous interactions between plants and leads to higher economic value per unit area, making it an important strategy in achieving sustainable agricultural development [15].

Soil microorganisms, as the crucial source of soil enzymes, are involved in soil biochemical reactions and cause beneficial changes in soil enzyme activities. Meanwhile, intercropping utilizes the relationship between biodiversity and ecosystem function; for example, the increase in biodiversity is related to the increase in plant primary productivity [16]. Cucumber planted in combination with garlic increased bacteria such as actinomycetes and decreased the fungi in rhizosphere soil [17]. Zhang *et al*. found that alfalfa/mulberry intercropping not only enriched the diversity of bacteria [18], but also changed the bacterial community structure; for example, the abundances of *Proteobacteria*, Actinomycetes and Firmicutes in mulberry intercropping, and *Proteobacteria*, Bacteroidetes, and Blastomonas in alfalfa intercropping were significantly higher than those in monoculture. Improving plant diversity directly increases the content and diversity of soil available carbon sources for microorganisms, which in turn increases their number, diversity, and activity, thereby inhibiting the invasion and reproduction of pathogenic bacteria [19, 20]. Jin *et al*. reported that mustard and sesame as green manure can improve the disease resistance of cucumber Fusarium wilt by changing bacterial community composition in cucumber rhizosphere soil [21]. Their results showed that the number of bacteria in watermelon rhizosphere soil increased, and those of *Fusarium oxysporum* and other fungi decreased after watermelon/upland rice intercropping, which significantly inhibited watermelon wilt disease [22]. Zhou *et al*. also found that cucumber/onion intercropping significantly improved soil bacterial diversity in cucumber rhizosphere soil [23]. Additionally, sugar, amino acids, and vitamins contained in crop root exudates can be used as main nutrients and energy for the survival and reproduction of rhizosphere microorganisms, which is conducive to the formation of corresponding rhizosphere microbial communities, thus improving their overall metabolic activity and promoting soil microbial diversity and health [24]. Furthermore, the changes in soil enzyme activities may be the result of the comprehensive effects of soil, water, air, heat, pH, microorganisms, organic matter, mineral nutrition and root exudates [25]. Contact among plant roots in intercropping systems can alter the dominant microbial species and communities [26, 27], form stable and diverse beneficial taxa, inhibit the growth and reproduction of harmful bacteria, and reduce the occurrence of soilborne diseases [22]. Therefore, it is of great significance to study the impact of companion planting on soil health status from the perspective of microorganisms.

Given the above, it is also imperative to determine whether soil rhizosphere microbes of companion plants are different from those of individually cultivated plants. Therefore, this study employed high-throughput amplicon sequencing technology to characterize and compare various soil representative enzymes and microbial compositions, as well as their symbiotic network in pepper monoculture and several companion planting systems.

## Materials and Methods

### Materials

Pepper (Hengjiao Co., China) was the main crop in this study, and companion crops included garlic, oat, cabbage, celery and white clover, which can be used to control pepper continuous cropping diseases, according to previous research. The fertilizers used were special organic fertilizers for fruits and vegetables (purchased from Ningxia Shangbonong Plant Protection Co., Ltd., China). The test soil was collected from a field under continuous cropping for 9 years in Xinji Township, Pengyang County, Ningxia Province, China. The soil properties were as follows: pH 8.53, total salt 3.22 g/kg, total nitrogen 0.12 g/kg, total P 0.89 g/kg, total K 39 g/kg, alkali-hydrolysed N 48 mg/kg, available P 40.66 mg/kg, available K 291 mg/kg, and organic matter 12.44 g/kg.

### Experimental Design and Sample Collection

The experiment was carried out at the Gongpeng Pepper Core Test Base in April, 2020, Xinji Township, Pengyang County, China. The test soil was mixed with organic fertilizer (v/10:1) and evenly loaded into 50 cm × 29 cm basins. Six treatments were set up: pepper monoculture (control, CK), companion garlic (T1), companion oat (T2), companion cabbage (T3), companion celery (T4), and companion clover (T5). After sprouting, pepper was sown in seed trays and planted in pots at the 4-leaf stage, and each pot had 1 plant. Five days after colonization, the seeds of companion plants were planted in a semicircle approximately 5 cm away from the pepper plant and according to the sowing amount, seed germination rate, and emergence time studied by predecessors. Six replicates (*i.e.*, 6 plots) were set for each treatment, which were randomly arranged and placed in the greenhouse for routine management. No chemical fertilizer was used during the trial period, but regular quantitative watering and manual weeding were carried out. After 50 days of companion treatment, the pepper rhizosphere soil was collected by the peeling separation method, and soil samples under the same treatment were mixed and divided into two parts, which were placed at 4°C and -80°C for enzyme activity determination and soil DNA extraction, respectively.

### Determination of Soil Enzyme Activities

Urease was determined by indophenol blue colorimetry, with urea as the substrate. After culture, the urease enzyme product ammonia (ignoring the ammonia nitrogen loss caused by nitrification) reacts with phenol and sodium hypochlorite in alkaline medium to produce blue indophenol. Catalase belongs to the haemoglobin enzyme family and contains iron, and it can also catalyze the decomposition of hydrogen peroxide into water and molecular oxygen; therefore, the enzyme activity can be determined according to the consumption of H_2_O_2_ or the production of O_2_, so it was determined with potassium permanganate titration. Sucrase determination was carried out by 3,5-dinitrosalicylic acid colorimetry according to the formation of colored compounds from the products of sucrose hydrolysis and certain substances (3,5-dinitrosalicylic acid) [28].

### DNA Extraction and High-Throughput Sequencing

Soil DNA extraction was performed by using the HiPure Soil DNA Kit (Omega, USA) according to the manufacturer’s instructions. After genomic DNA extraction, the bacterial 16S rRNA gene V3-V4 region was amplified using the primer pair P1 (341 F), 5¢-CCTACGGGNGGCWGCAG-3¢ and P2 (806R), 5¢-GGACTACHV GGGTATCTAAT-3¢, while the fungal ITS2 gene region was amplified using the primer pair P1 (ITS3_KYO_2_), 5¢-GATGAAGAACGYAGYRAA-3¢ and P2 (ITS4), 5¢-TCCTCCGCTTATTGATATGC-3¢. After amplicon purification and quantification, an Illumina NovaSeq 6000 platform was used for paired-end sequencing (PE250). The raw reads were deposited in the NCBI Sequence Read Archive (SRA) database (Accession Number: PRJCA010600).

### Microbial Community Analysis

Analysis of the raw data was conducted using FASTP (V 0.18.0), including read filtering and splicing, [29] and FLASH (version 1.2.11) [30]. QIIME (version 1.9.1) [31] was used to filter low-quality tags to obtain high-quality clean tags for subsequent analysis. The 16S rRNA and ITS gene sequences were clustered within operational taxonomic units (OTUs) at a 97% similarity level using UPARSE (version 9.2.64) [32]. The Ribosomal Database Project (RDP) Classifier (http://rdp.cme.msu.edu/) was used to analyze the taxonomy of each bacterial and fungal gene sequence against the Silva database (version 132) and Unite database (version 8.0) with a confidence threshold of 80% [33, 34]. A constrained principal coordinate analysis (PCoA) was performed using weighted UniFrac distances based on the OTU level. The bacterial and fungal community compositions were visualized using Circos software (http://circos.ca/). The linear discriminant analysis effect size (LEfSe) method was used to identify the most differentially abundant biomarker taxa among groups [35]. Alpha-diversity indices were calculated by QIIME [31]. To characterize the effects of companion planting on soil microbial co-occurrence patterns, a Spearman correlation matrix among soil bacterial and fungal genera was calculated using the “hmisc” and “igraph” packages. Strong correlations of bacterial and fungal genera with relative abundances greater than 0.1% (R > 0.7, *p* < 0.05) were retained for the visualization of networks using Gephi software [36-38]. Genera with the highest betweenness centrality scores were considered keystone taxa (top five genera), which have a unique and crucial role in microbial community and their deficiency can cause dramatic shifts in microbial structure and function.

### Statistical Analysis

One-way analysis of variance (ANOVA) with Duncan’s test was selected to analyze the differences in soil enzyme activities and α-diversity indices among all treatments. Analysis of similarity (ANOSIM) was performed to test whether the differences between groups were significantly greater than that within groups. Redundancy analysis (RDA) was performed to present the correlations between soil enzymes and microbial community structures using the vegan package in R with 999 permutations. Spearman correlation analysis was performed using IBM SPSS Statistics (v 25.0) (SPSS, USA). A *p*-value of < 0.05 was considered statistically significant.

## Results

### Influence of Companion Planting on Soil Enzyme Activities

The soil enzyme activities of pepper in monoculture and five companion systems are shown in Table 1. Compared with pepper monoculture, companion planting significantly increased soil urease (except for T5) and sucrase activities (*p* < 0.05), while soil catalase activity was significantly reduced (*p* < 0.05), and no significant differences were observed in the CK and T3 treatments.

### Soil Microbial Diversity and Community Structures

A total of 3,435,240 16S rRNA and 3,690,816 ITS quality sequences were obtained from all monoculture and companion soil samples, and the sequences were grouped into 4,825 bacterial and 542 fungal OTUs. The rarefaction curves (Fig. S1) for all samples indicated that the sequencing depth has reached an extent that covered all species.

**Fig. 1:**
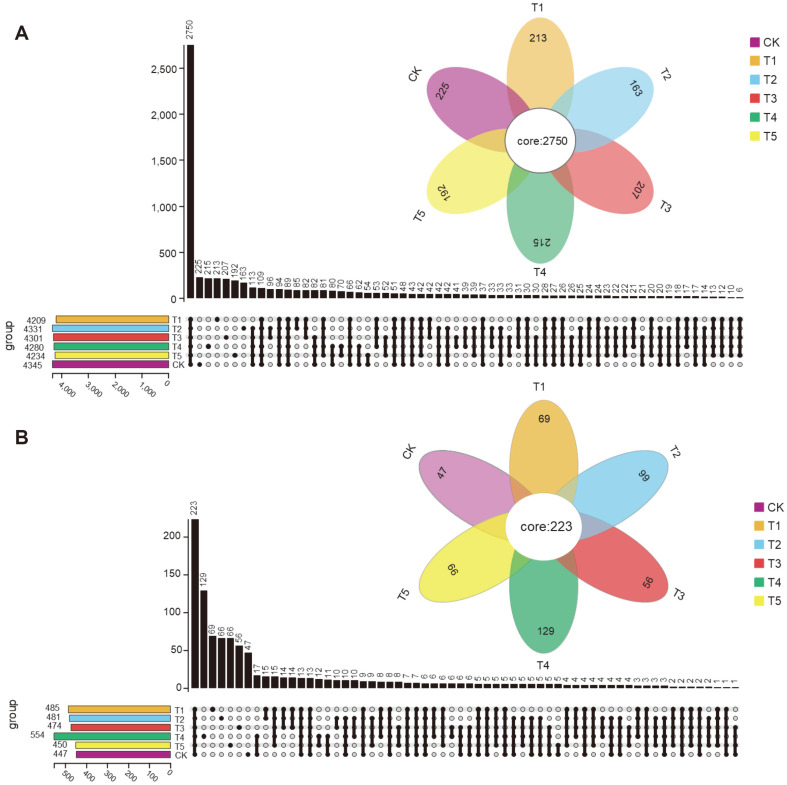
Shared OTU analysis in all treatments for bacteria (B) and fungi (F) based on Upset and petal plots. CK, pepper monoculture; T1, gallic companion cultivation; T2, oats companion cultivation; T3, cage companion cultivation; T4, celery cultivation; T5, white clover companion cultivation.

A petal plot was used to count the number of common and unique OTUs in all groups. As shown in Fig. 1, the numbers of common bacterial and fungal OTUs were 2,750 and 280, respectively, and the number of unique bacterial OTUs in the companion system was less than that in the monoculture system, while the result trends in the number for unique fungal OTUs were opposite. The soil bacterial and fungal richness and diversity are shown in Table 2. The number of soil bacterial OTUs was significantly lower in the T1 treatment than that in the control, while T2 significantly increased the bacterial Shannon index (*p* < 0.05). For fungi, the T1 and T3 treatments significantly increased the number of OTUs, and T2 treatment resulted in an increase in the Shannon index compared to CK (Table 2). The correlation analysis showed that the soil bacterial Shannon indices had no significant correlation with soil enzyme activities (*p* > 0.05), while fungal observed OTUs (r = 0.64, p =0.000; R = 0.55, *p* = 0.001) and Shannon indices (r = 0.43, *p* = 0.008; R = 0.52, *p* = 0.001) had significant positive correlations with urease and sucrase activities (Table 3).

**Fig. 2:**
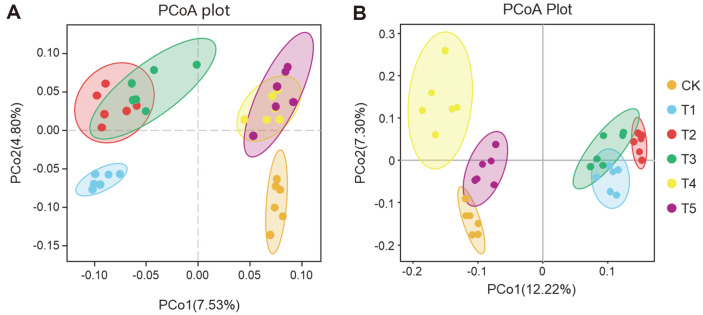
Principal co-ordinate analysis (PCoA) of bacterial (A) and fungal (B) communities in soils of the six treatments. Each point represents a sample. CK, pepper monoculture; T1, gallic companion cultivation; T2, oats companion cultivation; T3, cage companion cultivation; T4, celery cultivation; T5, white clover companion cultivation.

**Fig. 3:**
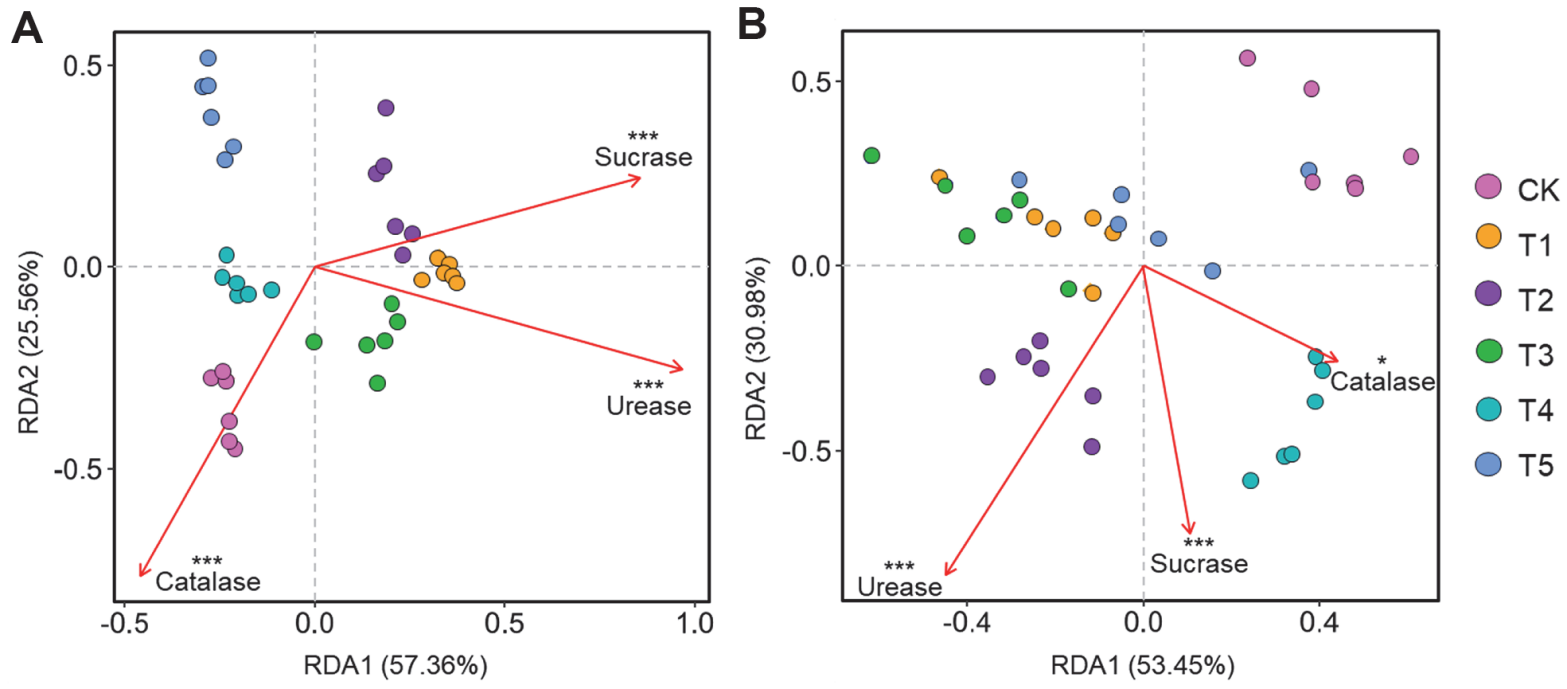
Redundancy analysis (RDA) of bacterial (A) and fungal (B) communities and soil enzyme activities for individual samples. Soil enzymes included urease, catalase and sucrase. The direction of the arrows indicates correlations with the first two canonical axes, and the length of the arrows represents the strength of the correlations.

PCoA was conducted to elucidate the shifts in the soil bacterial and fungal community structures in six treatments. PCoA plots revealed clear distinction in bacterial and fungal communities based on the OTUs level (Fig. 2, Table S1). The results showed that companion planting significantly changed soil microbial community structures, and there were significant differences among all treatments (except for T4-T5 fungi). The RDA showed that soil urease, catalase and sucrase activities were significantly correlated with bacterial and fungal community structures (*p* < 0.001) (Fig. 3, Table S2).

### Relative Abundances of Major Bacterial and Fungal Taxa

**Fig. 4:**
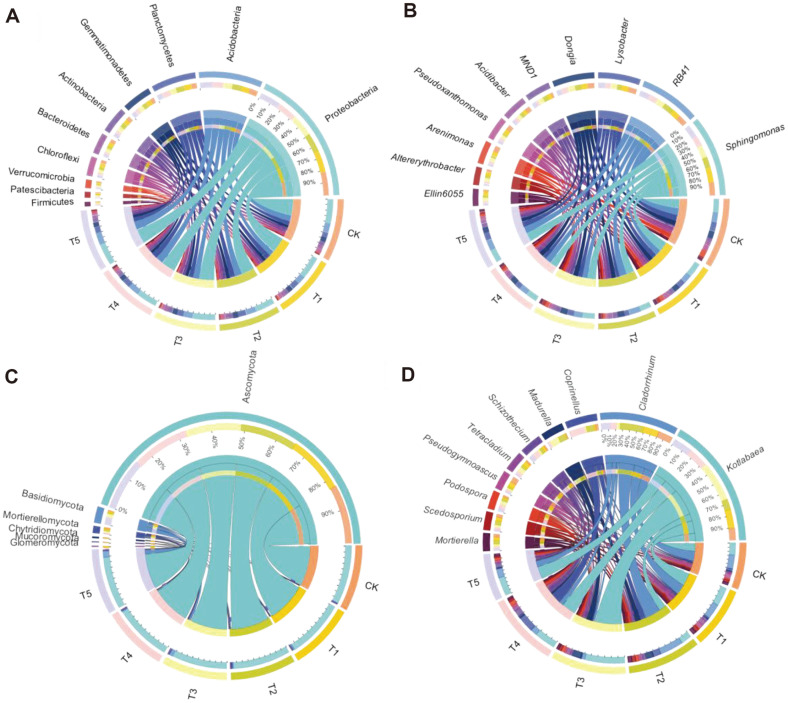
Bacterial (phylum (A) and genus (B)) and fungal (phylum (C) and genus (D)) community compositions at the phylum and genus levels in the six treatments. CK, pepper monoculture; T1, gallic companion cultivation; T2, oats companion cultivation; T3, cage companion cultivation; T4, celery cultivation; T5, white clover companion cultivation.

Companion planting treatments caused changes in the soil bacterial and fungal community compositions. The Circos plots show the composition and abundances of microbial communities at the phylum and genus levels in soil (Fig. 4, Tables S3 and S4). The top 10 bacterial phyla in all soil samples were *Proteobacteria*, Acidobacteria, Planctomycetes, Gemmatimonadetes, Actinobacteria, Bacteroidetes, Chloroflexi, Verrucomicrobia, Patescibacteria, and Firmicutes (Fig. 4A, Table S3), with relative abundances averaging 36.57, 17.04, 12.04, 7.83, 7.11, 5.55, 5.24, 2.23, 1.64, and 1.26%, respectively, and these 10 phyla accounted for 96.50% of all sequences. Companion planting treatments increased the relative abundances of Planctomycetes and Gemmatimonadetes, and decreased those of *Proteobacteria*, Bacteroidetes and Chloroflexi. The T1, T2 and T3 treatments caused the increases in the abundances of Actinobacteria and Paesciabacteria, which were reduced by the T4 and T5 treatments, while the abundance of Firmicutes in all treatments followed the opposite pattern compared to CK. In addition, there were no significant differences in the relative abundance of Verrucomicrobia in any treatment (*p* > 0.05). The taxonomical classification showed that 137 bacterial genera were detected at the genus level. *Sphingomonas*, RB41, *Lysobacter*, *Dongia*, MND1, *Acidibacter*, *Pseudoxanthomonas*, *Arenimonas*, *Altererythrobacter*, and *Ellin6055* were found to be the top 10 dominant bacterial genera (Fig. 4B, Table S3). Among the top 10 bacterial genera, companion planting treatments significantly increased the relative abundances of *MND1* and *Ellin6055*, and decreased those of *Lysobacter*, *Dongia*, *Acidibacter*, *Pseudoxanthomonas*, and *Arenimonas* compared to CK. Except for the T1 treatment, other companion planting treatments significantly reduced the relative abundance of *Sphingomonas*, while that of *Alterythrobacter* was significantly increased by the companion planting treatments (except for T5). Only the relative abundance of *RB41* had no significant difference in all treatments. In addition, 45 bacterial biomarkers were identified at the phylum, class, order, family, genus and species levels (LDA(log10) score > 3.5) (Fig. S3).

The fungal composition was dominated by Ascomycota, accounting for 93.23% of all sequences on average (Fig. 4C, Table S4). The subdominant phyla were Basidiomycota, Mortierellomycota, Chytridiomycota, Mucoromycota, and Glomeromycota. Significant differences were observed in the relative abundances of Ascomycota, Basidiomycota, Mortierellomycota, and Glomeromycota. Compared with monoculture, all companion treatments increased the relative abundances of Basidiomycota and Glomeromycota, and decreased that of Ascomycota. In addition, there were no significant differences in the abundances of Mortierellomycota, Chytridiomycota, and Mucoromycota. At the fungal genus level, *Kotlabaea*, *Cladorrhinum*, *Coprinellus*, *Madurella*, *Schizothecium*, *Tetracladium*, *Pseudogymnoascus*, *Podospora*, *Scedosporium*, and *Mortierella* were the top 10 dominant fungal genera (Fig. 4D, Table S4). Among the 10 genera, compared to monoculture, T4 significantly increased the relative abundances of *Kotlabaea*, *Madurella*, and *Tetracladium*, while T5 treatment increased those of *Coprinellus*, *Schizothecium*, and *Podospora*. T1 and T2 significantly increased the relative abundance of *Pseudogymnoascus*, but decreased that of *Podospora*. There were no significant differences in the abundances of *Scedosporium* and *Mortierella*. In addition, 98 biomarkers were found at the phylum, class, order, family, genus and species levels (with LDA(log10) score > 3.5) (Fig. S4).

### Co-Occurrence Network of the Soil Microbial Community

**Fig. 5:**
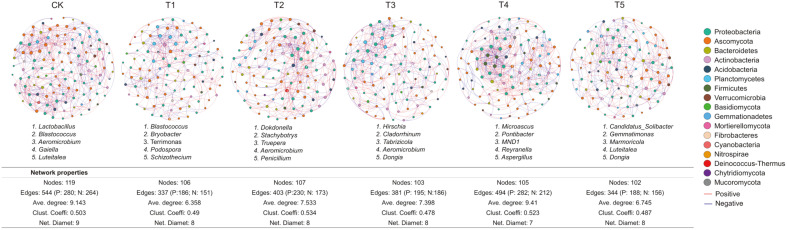
Connectedness among bacterial and fungal genera in the monoculture and companion cultivation groups based on network analysis. CK, pepper monoculture; T1, gallic companion cultivation; T2, oats companion cultivation; T3, cage companion cultivation; T4, celery cultivation; T5, white clover companion cultivation. Nodes (colored dots) indicate the genera involved in the networks and links indicate the relationship among the nodes. Red lines represent significant positive (Spearman’s correlation, R > 0.7 and *p* < 0.05) relationships, and blue lines denote negative (Spearman’s correlation, R < -0.7 and *p* < 0.05) relationships. The different colored dots represent the different phyla to which the genera belong. The numbers inside the nodes are the keystone genera (top five based on the betweenness centrality score) in the network.

Microbial co-occurrence network analysis was conducted to explore the co-occurrence patterns among bacteria and fungi at the genus level in soil. As shown in Fig. 5, 119 nodes and 544 edges were observed in the monoculture system (CK) network (R > 0.7, *p* < 0.05). Compared to CK, the numbers of nodes and edges in the five companion planting networks decreased, indicating that companion planting reduced the relationships among microorganisms, thus weakening the symbiotic patterns of the microbial community. In addition, the average degrees and clustering coefficients in the T3 and T5 treatments were lower than those of the control (CK), while the opposite results were observed in the T4 network. The network diameter in all companion treatments decreased compared to CK. Moreover, significant differences were also observed in the topological structure characteristics of the symbiosis network between control and companion planting treatments. Overall, companion planting treatments affected the network complexity of the microbial community. Additionally, there were differences in the keystone taxa in the microbial networks between the control and five companion planting treatments (Fig. 5). There were also significant differences in the positive and negative edges in the networks.

## Discussion

Soil biological activity, referring to the transformation, release, and fixation of soil nutrients, is an important indicator to assess soil fertility and health status [39]. However, plant diversity also has a great impact on the soil environment. This study revealed the impact of companion planting on soil biological activity.

Soil enzymes are involved in soil biochemical processes and play key roles in the occurrence, transformation, and availability of soil nutrients. They can reflect soil biological activity and biochemical reaction intensity, and are also sensitive to external environmental changes. Moreover, soil enzyme activities are important biological indicators of soil fertility, quality and health [40]; for example, they can transform complex soil organic matter into nutrients that can be directly used by plants and microorganisms [41]. Previous studies have shown that a reasonable intercropping mode can improve soil enzyme activities to varying degrees, which is conducive to the accumulation and transformation of soil nutrients [42]. The reason why plant diversity affects enzyme activities is that microorganisms have the potential to mineralize or fix essential nutrients by releasing enzymes into soil solution, and increase or reduce their availability to crops [42]. Soil catalase sensitively reflects the intensity of soil microbiological processes and crop metabolic processes to a certain extent [43]. Some studies have shown that soil catalase activity is closely related to root respiration intensity, and high root respiration intensity is an important factor for increasing the catalase activity [44]. In this study, catalase activity were significantly decreased by companion planting treatments, which can be attributed to the fact that companion plants did not develop pepper roots. After companion planting with pepper, root density and respiration intensity decreased, which inhibited the production of hydrogen peroxide. This result differs from the results of many companion planting studies and may have been caused by a lack of companion plants, and the companion period was shorter in our study, which was related to soil pH. Enzyme activity is directly affected by pH, and most soil enzymes show maximum activity at slightly acidic pH values [45]. Sucrase, also known as invertase, is a key enzyme of soil carbon metabolism, and its enzymatic reaction produces glucose, so in addition to being a nutrient source for plants and microorganisms it can also increase soluble nutrients in soil [46]. We found that companion treatments significantly increased soil sucrase activity, which promoted the metabolism of plants and microorganisms and further improved plant growth. Soil urease mainly comes from soil microorganisms that can promote the transformation of soil nitrogen into inorganic ammonia and carbonic acid, and its activity directly reflects soil nitrogen supply capacity [47]. In our study, except for T5 treatment, other companion planting treatments significantly increased urease activity, indicating that plants can promote microbial nitrogen metabolism and provide important nutrients for plants. This may have been due to the activation of rhizosphere microorganisms by microbial interactions and the resulting improvement of soil enzyme activities, which caused changes in soil nutrient contents that sped up the growth of plants.

Soil microbial diversity plays an important role in maintaining multiple functions in terrestrial ecosystems [48], and the increases in soil microbial diversity is conducive to soil function and health [49]. Several studies have noted that management measures to increase plant diversity often affect soil microbial communities [42, 50]. In this study, soil microbial diversity indices in companion planting were generally higher than that of monoculture. In addition, these indices were significantly different among the different treatments (Table 2), which is consistent with previous studies [51]. The improvement of soil microbial diversity by companion planting may be the reason why companion plants can increase pepper biomass [52]. Similar results were found in other studies, which also indicated that companion planting increased the microbial diversity of pepper continuous cropping soil and changed the soil microbial community structures. Companion planting systems play an important roles in the soil microbial diversity and community composition [53], and the reason may be due to the microbial interactions. In addition, the beta diversity showed that there were significant differences in microbial structures and compositions among different companion planting treatments (Fig. 2, Table S1), which may be caused by root exudates secreted by different plants. Similar results have been reported in previous soil microbial studies [54]. These results further confirmed the complexity from a micro perspective.

Intercropping has been shown to change soil microbial community compositions [55]. Changes in bacterial and fungal communities were mostly accompanied by differences in the abundances of specific phyla. In the present study, at the bacterial phylum level, the dominant bacterial taxa included *Proteobacteria*, Acidobacteria, Planctomycetes, Gemmatimonadetes, Actinobacteria, Bacteroidetes, Chloroflexi, Verrucomicrobia, Patescibacteria, and Firmicutes (Fig. 4A), suggesting that these microbial communities have high adaptability and play an important role in these ecosystems [56]. However, it was found that *Proteobacteria* was the dominant phylum in most studies [57, 58], which is the most abundant bacterial phylum in terrestrial habitats and is positively related to C mineralization [56]. In this study, companion planting led to increased relative abundances of Planctomycetes and Gemmatimonadetes and decreased those of *Proteobacteria* and Chloroflexi, which was inconsistent with the results of previous intercropping studies [57]. The reasons may be related to different soil properties and cultivation methods, and further study is needed. *Proteobacteria* feed on different stubborn carbon sources and strongly impact soil microbial community structures by providing functional capacity for the carbon cycle and more available nutrients for plant growth [59, 60]. As an important microorganism in the environment, Planctomycetes plays an important role in the biogeochemical cycle, such as in the carbon and nitrogen cycles [61, 62]. Our study found that companion planting significantly increased the relative abundance of Planctomycetes, indicating that plants can promote the metabolic cycle of main crops and stimulate plant growth. Therefore, the identification of this phylum may reflect the difference in the nitrogen fixation effect among different groups, indicating that companion planting may be an important way to improve the nitrogen fixation effect [4]. Ascomycota, Basidiomycota, Glomeromycota, and Mortierellomycota were detected as the dominant fungal phyla across all soil samples, in agreement with previous results [63]. Ascomycota, the most abundant group in the fungal phylum, is widely distributed in soil all over the world and can adapt to a variety of environments [64]. In addition, companion planting clearly shifted soil bacterial and fungal communities at the genus level, which is consistent with the previous research results, indicating that companion planting has an overall impact on microbial community composition. Pathogenic *Fusarium* was not the dominant fungus in this study, indicating that companion planting improves soil resistance and reduces its pathogenicity [65]. These results are of great significance to soil health status. To further understand the impact of the process mechanism of companion planting on pepper continuous cropping soil fertility and health, further basic research on functional genes is needed [56]. Meanwhile, our analysis of microbial diversity showed that there were differences in nitrogen fixation and other related microorganisms in different groups. Therefore, nutrient cycling and its microbial community are the biological basis for the improvements provided by companion planting [4].

Network analysis can be used to determine the interactions of microbial taxa in a niche and the keystone species that have the greatest impact on the microbial community [66]. A symbiotic network is an important factor affecting the stability of the microbial community under external interference. In the microbial networks obtained, the number of nodes and edges between bacterial and fungal genera both decreased in all of the companion systems compared to the monoculture system (Fig. 5), indicating that companion planting can reduce the competition between microorganisms, which may be because plant diversity improves nutrient availability and cycling. However, Pivato *et al*. [67] found that the effect of pea/wheat intercropping on rhizosphere bacterial network was not significant, which may be caused by the different planting methods as there are some differences between intercropping and companion planting. As one of the most common and effective planting methods, companion planting may have the ability to affect the symbiosis mode of soil microorganisms, and change it to a response mode of external interference. In this process, some synergistic microbial symbiosis modes may be generated to promote symbiosis and synergy among soil microorganisms, so further research in this area is needed.

In this study, RDA plots showed that soil enzyme activities were significantly correlated with soil microbial community structures, including urease, catalase, and sucrase (Fig. 3). In addition to enzyme activities, many studies have found that environmental factors such as pH, soil organic carbon, and available phosphorus determine soil microbial communities in different ecosystems [52, 68, 69]. The bacterial and fungal community structures were positively correlated with organic carbon, which can improve the soil environment by increasing soil carbon and reducing soil acidification to affect soil microbial communities. However, in companion planting systems, the environmental factors affecting soil microbial community structures need to be further studied.

To sum up, soil microbial community distribution patterns of pepper monoculture and companion plantings were compared using high-throughput sequencing technology. The results showed that companion planting significantly increased soil urease and sucrase activities, but decreased catalase activity compared to the monoculture system. In addition, companion planting improved the microbial diversity in pepper continuous cropping soil, and significantly changed soil microbial community structures and compositions. Soil enzyme activities were also found to be significantly correlated with microbial community structures. Moreover, the companion system reduced the complexity of microbial community networks, which may result in the provision of microbial nutrition and weaken the competition among microorganisms. These results should provide a theoretical basis and data for further research on reducing continuous cropping obstacles in agriculture.

## Supplemental Materials

Supplementary data for this paper are available on-line only at http://jmb.or.kr.

## Figures and Tables

**Table 1:** Soil enzyme activity analysis.

Treatments	Urease (mg/g/24 h)	Catalase (mg/g/min)	Sucrase (mg/g/24 h)
CK	1.53 ± 0.01 E	1.43 ± 0.01 A	17.61 ± 0.69 F
T1	3.18 ± 0.01 A	1.23 ± 0.01 D	58.37 ± 0.55 A
T2	2.23 ± 0.01 C	1.16 ± 0.01 E	31.59 ± 0.35 C
T3	2.44 ± 0.04 B	1.42 ± 0.02 A	44.84 ± 0.68 B
T4	1.82 ± 0.01 D	1.33 ± 0.01 B	21.40 ± 0.60 E
T5	1.22 ± 0.02 F	1.27 ± 0.01 C	30.06 ± 0.48 D

^a^CK, pepper monoculture; T1, gallic companion cultivation; T2, oats companion cultivation; T3, cage companion cultivation; T4, celery cultivation; T5, white clover companion cultivation.

^b^The mean value ± SD (*n* = 6). Different letters in the same column represent significant differences at the *p* = 0.05 level.

**Table 2:** Community diversity indices of bacteria and fungi (at 97% sequence similarity) based on the 16S and ITS rRNA genes.

Treatments	Bacteria		Fungi	

	Observed OTU	Shannon	Observed OTU	Shannon
CK	4742 ± 112 A	10.23 ± 0.06 BC	423 ± 29 BC	4.76 ± 0.34 BC
T1	4610 ± 85 B	10.21 ± 0.06 C	474 ± 20 A	5.16 ± 0.11 AB
T2	4669 ± 99 AB	10.31 ± 0.05 A	457 ± 18 AB	5.34 ± 0.28 A
T3	4640 ± 94 AB	10.28 ± 0.07 ABC	466 ± 34 A	5.09 ± 0.30 AB
T4	4692 ± 93 AB	10.25 ± 0.06 ABC	457 ± 44 AB	4.63 ± 0.33 C
T5	4655 ± 71 AB	10.30 ± 0.02 AB	407 ± 32 C	4.84 ± 0.45 BC

^a^CK, pepper monoculture; T1, gallic companion cultivation; T2, oats companion cultivation; T3, cage companion cultivation; T4, celery cultivation; T5, white clover companion cultivation.

^b^The mean value ± SD (*n* = 6). Different letters in the same column represent significant differences at the *p* = 0.05 level.

**Table 3:** Spearman correlation analysis of soil microbial diversity and enzyme activities.

Soil enzyme	Bacteria		Fungi	

	Observed OTU	Shannon	Observed OTU	Shannon
Urease	-0.28	-0.23	0.64**	0.43**
Catalase	0.21	-0.20	-0.15	-0.38*
Sucrase	-0.388*	-0.04	0.55**	0.52**

*p < 0.05 and **p < 0.01.
