# Supplementary material for: Influence of Companion Planting on Microbial Compositions and Their Symbiotic Network in Pepper Continuous Cropping Soil
Source: J Microbiol Biotechnol. 2023 Mar 31;33(6):760–70. doi: 10.4014/jmb.2211.11032 (PMC10331949; doi:10.4014/jmb.2211.11032)
Supplement: Supplementary file 1 [file jmb-33-6-760-supple.pdf]

J. Microbiol. Biotechnol.
https://doi.org/10.4014/jmb.2211.11032

Supplementary Tables and Figures

Influence of Companion Planting on Microbial Compositions and Their Symbiotic Network in Pepper Continuous Cropping Soil

Jingxia Gao, Fengbao Zhang*

Institute of Soil and Water Conservation, Northwest A&F University, Yangling 712100, China

Prof. Fengbao Zhang

Institute of Soil and Water Conservation,

Northwest A&F University, Yangling, 712100, China

E-mail: xiehua@0002163.com

Table S1. The differences in the bacterial and fungal community structures among all treatments. The results were analyzed using ANOSIM analysis. *p*-value < 0.05 (*) and *P*-value < 0.01 (**) represent the significant difference between the two treatments. CK indicates pepper monoculture, T1 indicates gallic companion cultivation, T2 indicates oats cultivation, T3 indicates cage cultivation, T4 indicates celery cultivation, and T5 indicates white clover cultivation.

Group	Bacteria		Fungi	
	R statistic	*P*-value	R statistic	*P*-value
CK-T1	1	0.002**	0.9611	0.003**
CK-T2	0.955	0.002**	0.9111	0.005**
CK-T3	0.9813	0.003**	0.9722	0.004**
CK-T4	0.9278	0.004**	0.768	0.003**
CK-T5	0.9815	0.003**	0.3664	0.006**
T1-T2	0.6878	0.002**	0.5148	0.001**
T1-T3	0.8133	0.005**	0.8444	0.002**
T1-T4	0.9944	0.003**	0.9973	0.002**
T1-T5	1	0.002**	0.8505	0.002**
T2-T3	0.2977	0.01*	0.8389	0.005**
T2-T4	0.9286	0.001**	0.9787	0.002**
T2-T5	0.9378	0.001**	0.8307	0.003**
T3-T4	0.9493	0.003**	0.984	0.005**
T3-T5	0.9867	0.002**	0.8082	0.001**
T4-T5	0.7426	0.004**	0.1963	0.069

Table S2. The results of Monte Carlo permutation test in the RDA (using function permutest and envfit in the vegan package of R). r^2 represents the coefficients of determination of soil properties that were related to OTUs. The value is the cosine value of the angle between the arrow and the axis (RDA1 and RDA2).

**Bacteria**				
**Test: F= 3.3515, *P* = 0.001*** **				
Soil enzymes	RDA1	RDA2	r^2	*P* value
Urease	0.97903	-0.20370	0.8992	0.001***
Catalase	-0.55259	-0.83345	0.7248	0.001***
Sucrase	0.96332	0.26837	0.7321	0.001***
**Fungi**				
**Test: F= 4.9235, *P* = 0.001*** **				
Soil enzymes	RDA1	RDA2	r^2	*P* value
Urease	-0.42579	-0.90482	0.7634	0.001***
Catalase	0.82091	-0.57106	0.1763	0.043*
Sucrase	0.06025	-0.99818	0.4451	0.001***

Table S3. The relative abundances of top 10 phyla and genera for bacteria. The mean value ± standard deviation (n = 6). Different letters in the same column represent significant differences at the $p = 0.05$ level. CK indicates pepper monoculture, T1 indicates gallic companion cultivation, T2 indicates oats cultivation, T3 indicates cage cultivation, T4 indicates celery cultivation, and T5 indicates white clover cultivation.

		**CK**	**T1**	**T2**	**T3**	**T4**	**T5**
Phylum	Proteobacteria	41.03±2.84a	37.69±1.68b	32.89±2.79c	37.27±3.30b	34.68±3.83bc	35.88±1.27bc
	Acidobacteria	15.50±1.80bc	13.77±0.97c	17.13±1.76b	15.36±1.23bc	19.44±1.52a	21.02±1.64a
	Planctomycetes	9.50±2.33b	12.91±3.04ab	13.20±4.06ab	12.48±3.88ab	14.18±4.84a	9.94±0.54ab
	Gemmatimonadetes	6.87±0.60c	8.30±0.53a	8.59±0.55a	8.09±0.58ab	7.51±0.50b	7.62±0.17b
	Actinobacteria	6.83±0.73c	7.34±0.33bc	8.60±1.44a	7.95±0.82ab	5.30±0.69d	6.61±0.31c
	Bacteroidetes	6.35±0.13a	5.96±0.60a	5.18±0.83b	6.21±0.53a	4.50±0.52b	5.10±0.47b
	Chloroflexi	5.93±0.59a	5.52±0.22a	5.69±0.62a	4.89±0.28b	4.44±0.53b	4.97±0.25b
	Verrucomicrobia	2.10±0.70a	2.22±0.73a	2.63±0.55a	2.32±0.50a	1.89±0.54a	2.19±0.46a
	Patescibacteria	1.57±0.16b	2.07±0.16a	1.60±0.18b	1.69±0.20b	1.48±0.21b	1.45±0.16b
	Firmicutes	1.03±0.15b	0.96±0.24b	0.98±0.24b	0.61±0.12b	2.89±0.10a	1.10±0.25b
Genus	*Sphingomonas*	3.62±0.36ab	3.90±0.59a	3.09±0.40bc	3.29±0.44bc	2.94±0.56c	3.56±0.34ab
	RB41	2.70±0.44a	2.19±0.31a	3.04±1.50a	2.29±0.41a	2.44±0.39a	2.74±0.40a
	Lysobacter	2.32±0.32a	1.96±0.24ab	1.33±0.22c	1.68±0.36bc	1.44±0.47c	2.04±0.51ab
	Dongia	1.95±0.24a	1.65±0.13bc	1.81±0.32ab	1.93±0.18a	1.92±0.22a	1.41±0.12c
	MND1	0.63±0.05c	1.07±0.16b	1.44±0.23a	1.40±0.15a	0.75±0.08c	1.19±0.18b
	Acidibacter	1.40±0.08a	0.87±0.08cd	1.16±0.23b	0.99±0.15cd	1.03±0.10bc	0.85±0.11d
	Pseudoxanthomonas	1.46±0.50a	1.31±0.19a	0.55±0.22b	0.86±0.22b	0.71±0.28b	0.89±0.34b
	Arenimonas	1.72±0.31a	1.11±0.13b	0.56±0.08e	0.92±0.28bc	0.81±0.13cd	0.67±0.15de
	Altererythrobacter	0.67±0.06bc	0.94±0.09a	0.75±0.13b	0.89±0.07a	0.71±0.06b	0.60±0.08c
	Ellin6055	0.63±0.08c	0.94±0.07a	0.73±0.05bc	0.79±0.16b	0.66±0.08c	0.67±0.04c

Table S4. The relative abundances of phyla and top 10 genera for fungi. The mean value ± standard deviation (n = 6). Different letters in the same column represent significant differences at the *p* = 0.05 level. CK indicates pepper monoculture, T1 indicates gallic companion cultivation, T2 indicates oats cultivation, T3 indicates cage cultivation, T4 indicates celery cultivation, and T5 indicates white clover cultivation.

		**CK**	**T1**	**T2**	**T3**	**T4**	**T5**
Phylum	Ascomycota	96.99±1.37a	95.74±1.90a	94.41±1.67a	90.86±7.44ab	95.90±2.65a	85.49±14.79b
	Basidiomycota	0.66±0.36b	2.22±2.23b	1.90±1.14b	5.75±6.91ab	1.65±0.91b	11.31±1.02a
	Mortierellomycota	1.45±0.85a	1.59±0.83a	2.16±1.05a	2.34±2.80a	1.39±1.44a	1.33±1.04a
	Chytridiomycota	0.07±0.04a	0.12±0.08a	0.95±1.86a	0.19±0.17a	0.08±0.07a	0.06±0.06a
	Mucoromycota	0.02±0.02a	0.03±0.02a	0.04±0.03a	0.02±0.01a	0.39±0.70a	0.12±0.08a
	Glomeromycota	0.00±0.00c	0.01±0.01bc	0.08±0.04a	0.07±0.03a	0.02±0.01bc	0.04±0.02b
Genus	*Kotlabaea*	11.96±4.96bc	7.51±4.02bc	11.97±3.97bc	6.91±3.30c	25.75±6.43a	13.54±5.95b
	Cladorrhinum	5.87±.0.74bc	9.17±4.62abc	10.02±5.86ab	15.14±4.17a	2.03±0.25c	4.25±2.07bc
	Coprinellus	0.32±0.03b	1.57±0.31b	0.88±0.13b	4.96±0.69ab	0.65±0.08b	10.29±5.18a
	Madurella	0.34±0.03c	0.42±0.03c	0.30±0.17c	4.87±0.57ab	5.71±0.56a	1.55±0.50bc
	Schizothecium	2.84±0.67b	0.67±0.19d	1.65±0.34cd	1.81±0.30c	1.46±0.38cd	4.21±1.85a
	Tetracladium	0.77±0.19b	0.73±0.06b	3.12±1.81a	1.66±0.12ab	2.90±0.31a	2.22±1.21ab
	Pseudogymnoascus	1.03±0.32b	3.29±0.35a	3.08±1.03a	1.11±0.24b	0.88±0.22b	1.38±0.30b
	Podospora	1.83±0.25b	0.80±0.18c	0.62±0.22c	2.06±0.80b	2.30±0.50ab	3.01±1.25a
	Scedosporium	1.07±0.08a	1.65±0.38a	2.31±1.27a	2.50±0.26a	1.70±0.73a	1.36±0.42a
	Mortierella	1.45±0.09a	1.59±0.83a	2.16±1.05a	2.34±0.28a	1.39±0.14a	1.32±0.45a

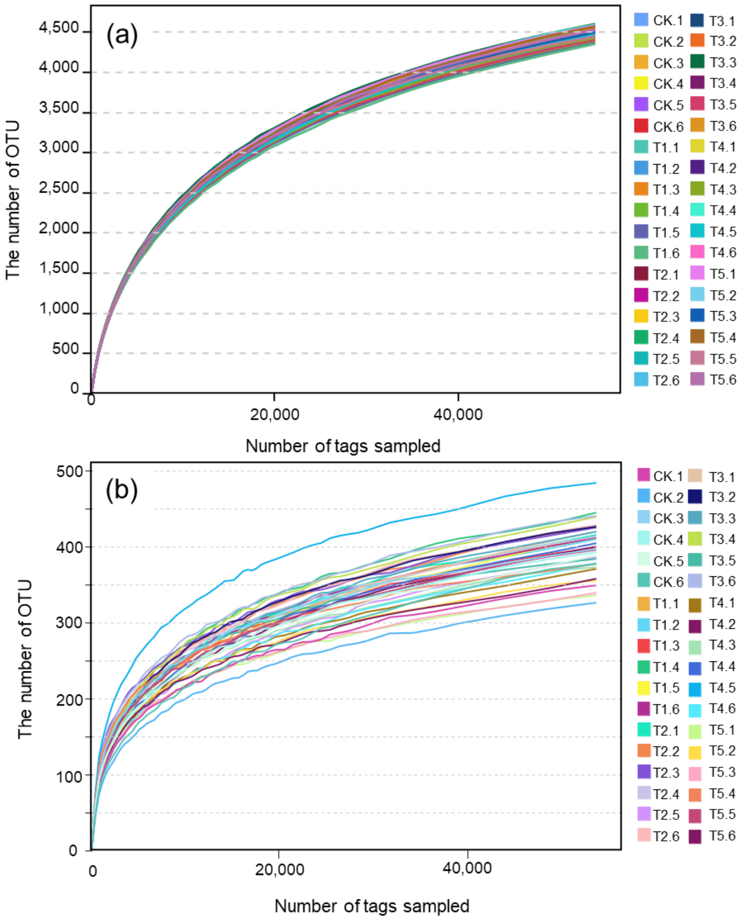

Figure S1. Rarefaction curves of 16S rRNA gene (A) and ITS gene (B).

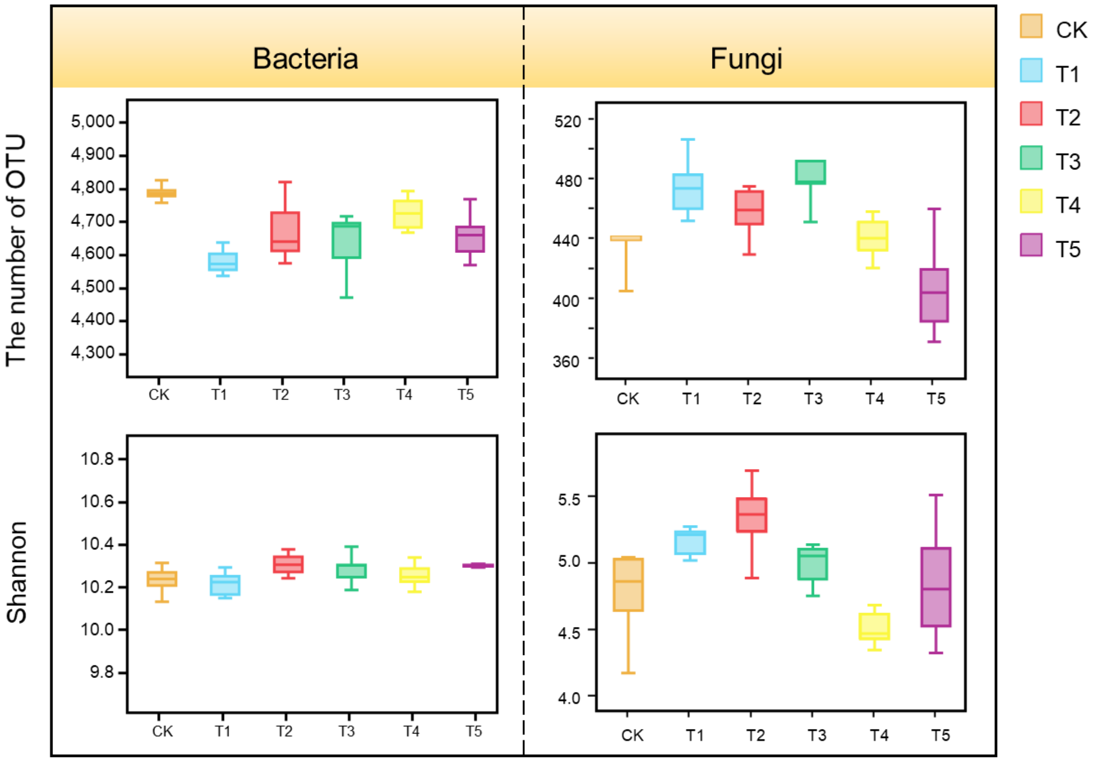

Figure S2. Comparison of microbial diversity indices among six treatments.

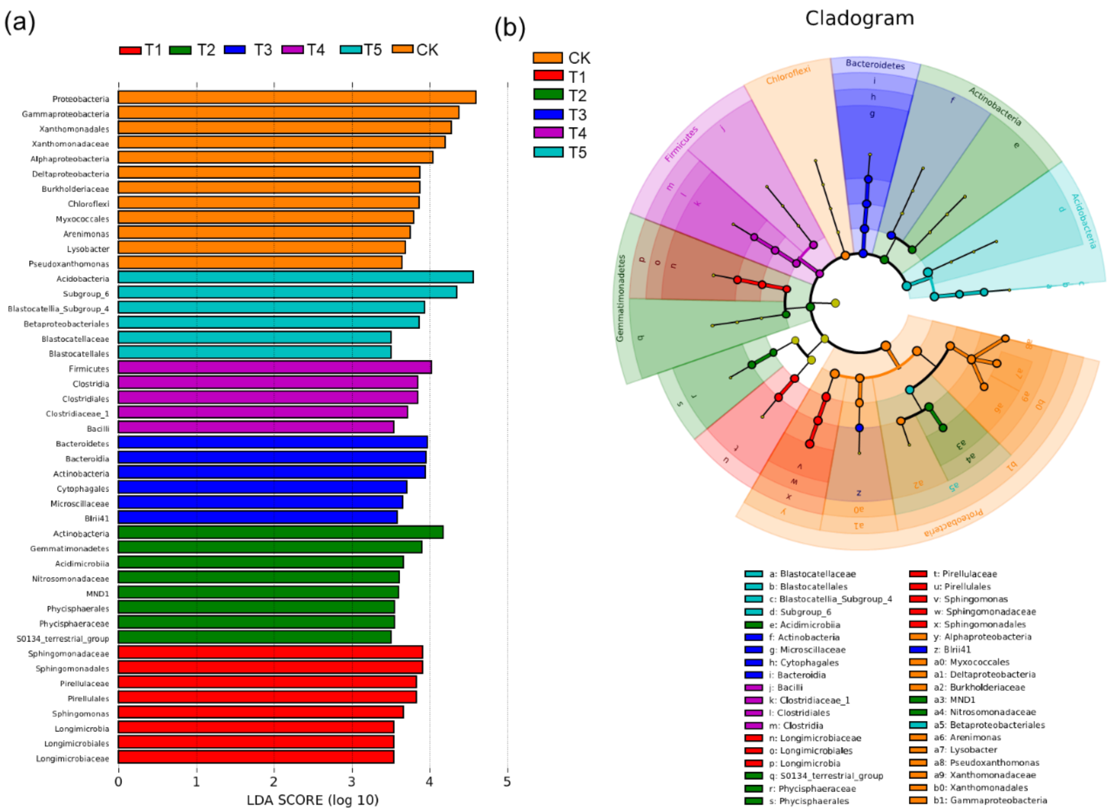

Figure S3. Results of LEfSe analysis showing taxa that significantly differed in the six treatments (A). Cladogram plotted from LEfSe analysis showing the significant differences ($p < 0.05$) in relative abundance of bacterial taxon among five treatments (B).

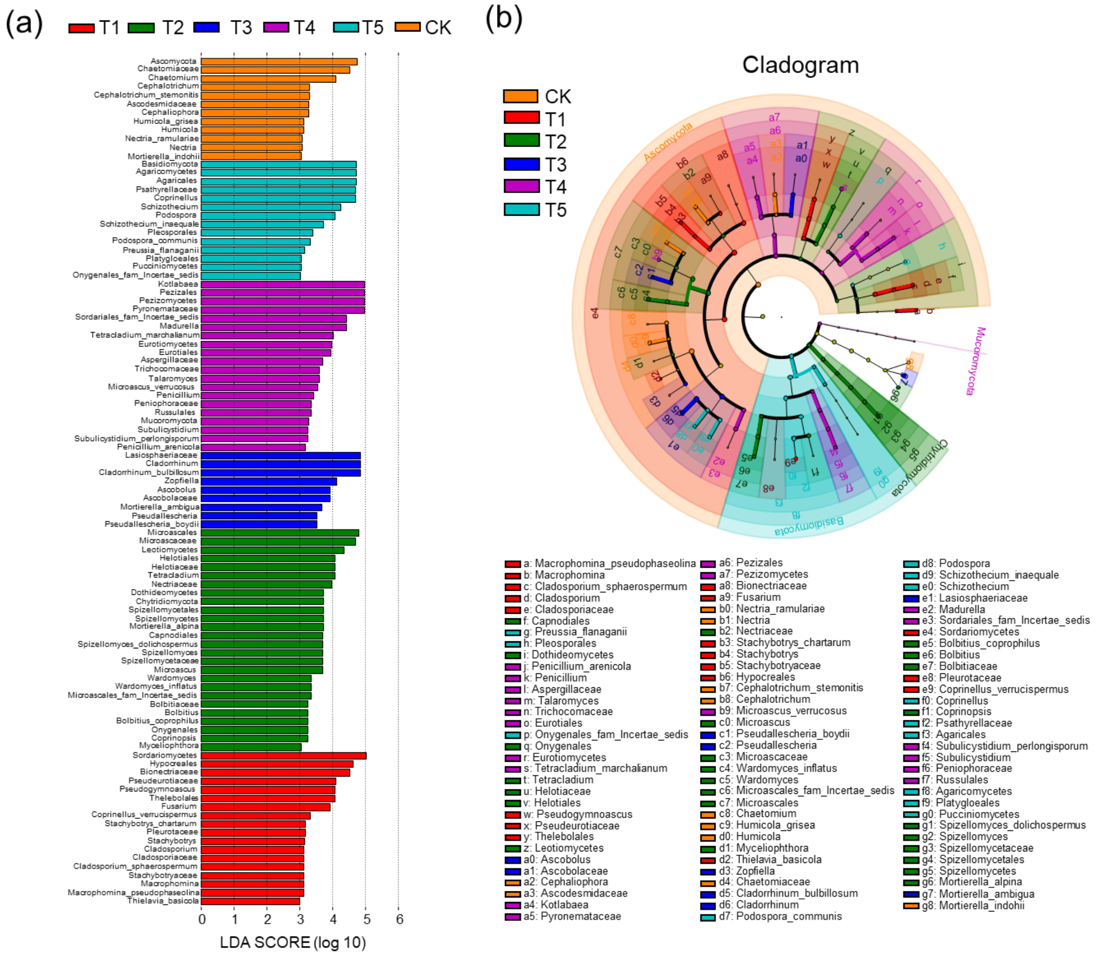

Figure S4. Results of LEfSe analysis showing fungal taxa that significantly differed in the five treatments (A). Cladogram plotted from LEfSe analysis showing the significant differences ($p < 0.05$) in relative abundance of fungal taxon among six treatments (B).
